# Crosstalk between neutrophil extracellular traps and gut microbiota in ulcerative colitis: traditional Chinese medicine strategies

**DOI:** 10.3389/fcimb.2025.1692312

**Published:** 2025-10-15

**Authors:** Yiyi Feng, Yuchen Liu, Xiuxiu Qiu, Jianfang Jiang, Jianling Mo, Yichuan Xv

**Affiliations:** ^1^ Department of Traditional Chinese Medicine, Sir Run Run Shaw Hospital, Zhejiang University School of Medicine, Hangzhou, Zhejiang, China; ^2^ Department of Gastroenterology, Longhua Hospital, Shanghai University of Traditional Chinese Medicine, Shanghai, China; ^3^ Department of Oncology, Longhua Hospital, Shanghai University of Traditional Chinese Medicine, Shanghai, China; ^4^ Department of Nursing, Sir Run Run Shaw Hospital, Zhejiang University School of Medicine, Hangzhou, Zhejiang, China

**Keywords:** ulcerative colitis, gut microbiota, neutrophil extracellular traps, traditional Chinese medicine, inflammation

## Abstract

Ulcerative colitis (UC), a chronic and complex inflammatory bowel disorder, presents ongoing therapeutic challenges. Although multi-tiered anti-inflammatory strategies represent significant advances, issues like treatment resistance and adverse effects persist. Consequently, identifying more effective therapeutic targets and potentially curative strategies remains imperative. Emerging evidence underscores neutrophils, particularly through neutrophil extracellular trap (NET) formation, as pivotal contributors to UC pathogenesis. In affected individuals, excessive NET accumulation exacerbates intestinal inflammation, compromises the epithelial barrier, activates coagulation pathways, promotes resistance to biologic therapies, and may even facilitate malignant transformation. Critically, a bidirectional interplay exists between NETs and the gut microbiota (GM) in this disease. Recent research indicates that certain traditional Chinese medicine (TCM) herbal extracts and formulas hold promise for modulating aberrant NET generation and GM composition. This review examines the roles of NETs and GM in UC pathogenesis and synthesizes evidence on potential TCM-based interventions targeting these pathways, offering novel perspectives for future therapeutic development.

## 1 Introduction

Ulcerative colitis (UC) is a chronic inflammatory disorder characterized by recurring mucosal inflammation in the colon (Ungaro et al., 2017). Affected individuals experience recurrent bloody diarrhea, abdominal pain, and tenesmus, severely compromising quality of life. Current management employs aminosalicylates, glucocorticoids, immunomodulatory agents, and biologic therapies. Despite these advances, roughly 40% of patients fail to respond to anti-tumor necrosis factor (TNF)-α therapy (Gorelik et al., 2022). Moreover, prolonged biologic use elevates infection and malignancy risks (Kobayashi et al., 2021). This highlights the critical need for deeper mechanistic understanding and novel therapeutic approaches for UC.

The activation of neutrophils, especially the formation of neutrophil extracellular traps (NETs), is increasingly recognized in UC pathogenesis (Dos Santos Ramos et al., 2021). These structures, composed of decondensed chromatin and antimicrobial proteins, are extruded by neutrophils to capture pathogens. Excessive NET generation can cause tissue damage and exacerbate inflammation (Herre et al., 2023; Dos Santos Ramos et al., 2021). Additionally, NETs mediate processes like immuno-thrombosis (Skendros et al., 2020), contribute to biologic therapy resistance (Curciarello et al., 2020), and play a role in inflammation-associated carcinogenesis (Van Der Windt et al., 2018). However, neutrophil-targeting methods have not achieved satisfying efficacy in UC (Danne et al., 2024). Dysbiosis of the gut microbiota (GM) remains a crucial factor in UC development (Wang et al., 2023a). The microbiota interacts with the immune system, influencing neutrophil generation, maturation, and activation (Wang et al., 2023a). Therefore, targeting this crosstalk to modulation NET formation may offer novel therapeutic avenues for improving UC clinical outcomes.

The integration of traditional Chinese medicine (TCM) into UC management has gained significant interest due to its multifaceted therapeutic mechanisms. Clinical evidence confirms that specific herbal formulations effectively induce clinical remission and promote mucosal healing in UC patients (Shen et al., 2021; Gong et al., 2012; Sugimoto et al., 2016). Further research revealed that these therapeutic outcomes are attributed to the modulation of GM composition, restoration of intestinal barrier integrity, and regulation of immune responses (Li et al., 2022a; Wei et al., 2021; Yan et al., 2018). The crosstalk between NETs and microbiota represents a key focus in UC research, receiving growing attention in TCM studies. Deciphering TCM's regulatory effects on NETs and gut flora in UC may advance targeted therapeutic development.

This review emphasizes the role of NETs and GM in UC, alongside TCM interventions targeting this crosstalk. We aim to offer actionable perspectives for advancing TCM-based strategies that modulate these targets to optimize UC management.

## 2 Neutrophils in ulcerative colitis: an overview

Neutrophils are integral to the pathophysiology of UC, with peripheral neutrophil counts significantly higher in active UC patients compared to healthy controls or those in remission (Bamias et al., 2022).Elevated neutrophil counts correlate with poor responses to biologic therapies and a worse prognosis. The neutrophil-to-lymphocyte ratio (NLR) before treatment has been linked to clinical relapse following tacrolimus induction in UC patients (Nishida et al., 2019). A cohort study found that an elevated NLR can predict relapse even in patients with mucosal healing (Kurimoto et al., 2023). Other studies have identified additional neutrophil-related indicators for predicting UC activity. The neutrophil-to-bilirubin ratio is positively associated with disease activity, with lower ratios seen in those who achieve mucosal healing (Huang et al., 2023). Another study suggested that the neutrophil-to-albumin ratio might predict clinical outcomes and long-term prognosis in UC patients treated with infliximab (IFX) (Zhang et al., 2024). A study on newly diagnosed, treatment-naive UC patients found that differentially expressed genes were primarily enriched in neutrophil-related pathways, such as chemotaxis, activation, and degranulation (Juzenas et al., 2022).The C-X-C motif chemokine receptor 1/2 (CXCR1/2) genes are central to the co-expression module in UC, with their expression levels significantly correlating with clinical indicators like albumin and C-reactive protein (Juzenas et al., 2022).

Excessive neutrophil recruitment to the colonic mucosa is a hallmark of UC. Upon activation, these infiltrating neutrophils release various pro-inflammatory mediators, such as reactive oxygen species (ROS), myeloperoxidase (MPO), matrix metalloproteinases (MMPs), and neutrophil elastase (NE), which are central to tissue damage and the inflammatory process in the affected mucosa (Kolaczkowska and Kubes, 2013). ROS cause cellular injury by damaging cell membranes and activating inflammatory pathways, while MMPs and NE degrade cell junctions, leading to crypt distortion and abscess formation, typical of UC pathology (Kang et al., 2022). Fecal calprotectin (FC), a calcium-binding protein dimer found in neutrophils, is a reliable biomarker for intestinal inflammation (Tibble et al., 2000). FC levels below 100 μg/g have shown ≥85% accuracy in predicting histological remission (Singh et al., 2024). A multicenter study found that histological assessment of neutrophil infiltration can predict long-term UC outcomes, including the need for treatment adjustments, colectomy, or biologic therapy escalation (Parigi et al., 2023). A large cohort study also revealed that persistent neutrophil infiltration at week 14 is linked to failure in achieving endoscopic and histological healing by week 52 in UC patients receiving biologic treatments (Narula et al., 2022).

## 3 Roles of neutrophil extracellular traps in ulcerative colitis

### 3.1 Formation-clearance imbalance of neutrophil extracellular traps

**Figure 1:**
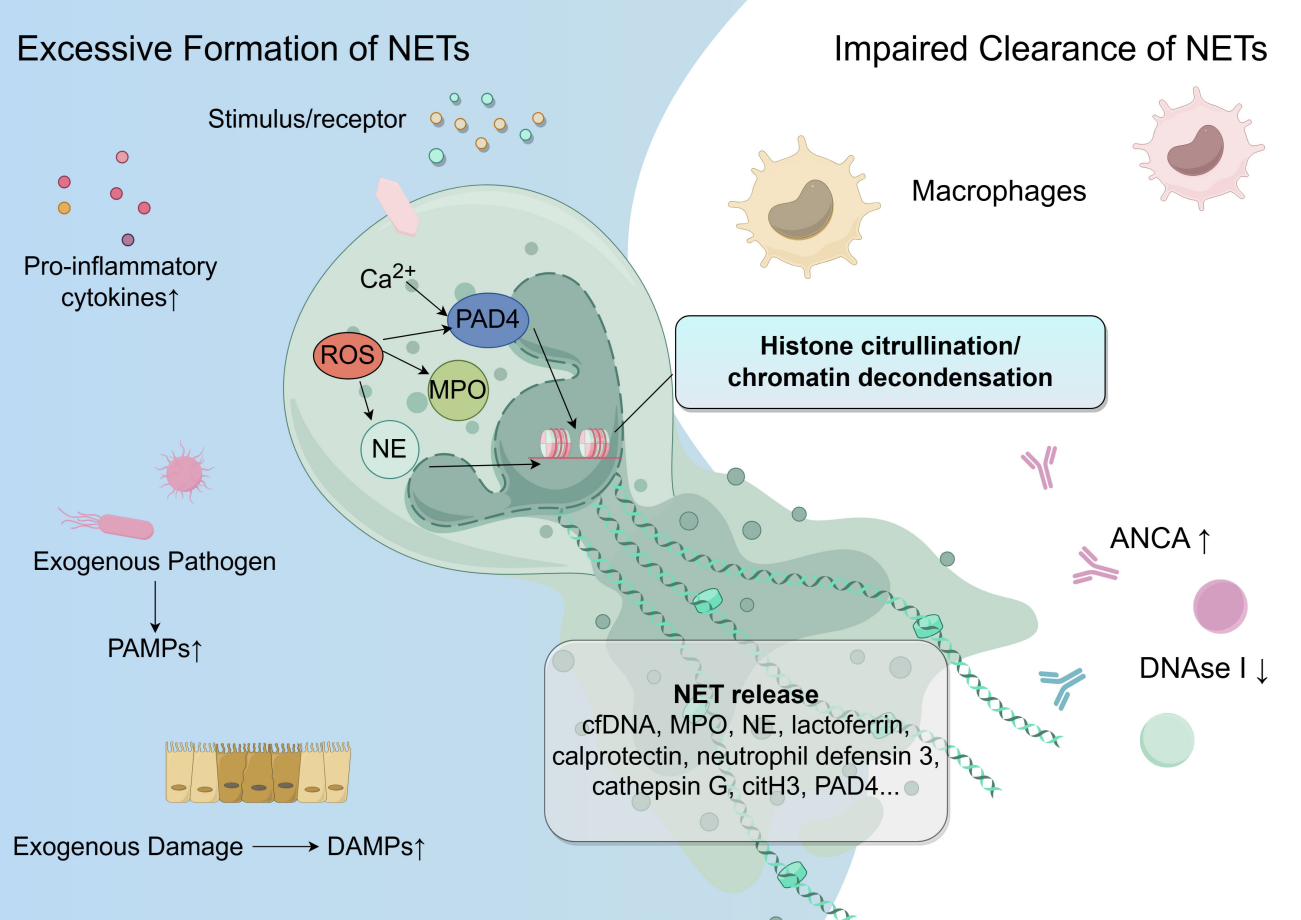
The unbalance of NET formation and clearance in UC. The accumulation of NETs is primarily due to an imbalance between NET formation and clearance. Increased NET formation is potentially triggered by proinflammatory cytokines, DAMPs, and PAMPs. These stimuli interact with receptors on neutrophils, leading to elevated intracellular calcium, NADPH, and ROS. This is followed by the translocation of NE, MPO, and PAD4, which collectively induce histone citrullination, chromatin decondensation, and DNA extrusion. Simultaneously, NET degradation capacity is reduced due to decreased levels of DNase I. Additionally, the development of ANCAs in UC may serve as a protective factor against NET degradation. NET, neutrophil extracellular trap; DAMP, damage-associated molecular pattern; PAMP, pathogen-associated molecular pattern; ROS, reactive oxygen species; NE, neutrophil elastase; MPO, myeloperoxidase; PAD, peptidylarginine deiminase; ANCA, antineutrophil cytoplasmic antibodies; UC, ulcerative colitis; cfDNA, cell-free DNA; citH3, citrullinated histone H3.

NETs, first identified in 2004 (Brinkmann et al., 2004), are an important part of the neutrophil response. NET formation, which can be either lytic or non-lytic, is triggered by various stimuli (Long et al., 2024). NETs are composed of DNA, citrullinated histones, and granule proteins such as MPO, NE, cathepsin G, and MMP-9 (Boeltz et al., 2019). The process begins with the activation of NADPH, ROS, and increased intracellular calcium, leading to the translocation of NE, MPO, and peptidylarginine deiminase-4 (PAD4), which results in histone citrullination, chromatin decondensation, and DNA extrusion (Long et al., 2024). NETs help immobilize and remove pathogens, but in pathological conditions, they can exacerbate inflammation and contribute to tissue damage (Schoen et al., 2022). The balance between NET formation and clearance is crucial for maintaining homeostasis, and when disrupted, it can lead to disease development (See **Figure 1**).

Emerging evidence indicates a heightened formation of NETs in UC. Multiple techniques such as immunohistochemistry and western blotting have all consistently shown elevated levels of NET-associated components in intestinal mucosa or fecal samples, such as cell-free DNA, MPO, NE, lactoferrin, calprotectin, neutrophil defensin 3, cathepsin G, citrullinated histone H3, and complexes of DNA, MPO, and NE (Lehmann et al., 2019; Bennike et al., 2015; Li et al., 2020). Notably, PAD4 expression is increased in UC colon tissues (Leppkes et al., 2022), with PAD4 being crucial for NET formation by catalyzing histone H3 citrullination. Additionally, CD177^+^ (Zhou et al., 2018) and CCR5^+^ (Neuenfeldt et al., 2022) neutrophils are more prevalent in the blood and inflamed mucosa of UC patients, as these cells are known to be more prone to NET generation.

NET formation can be triggered by damage-associated molecular patterns (DAMPs), pathogen-associated molecular patterns (PAMPs) and cytokines. DAMPs, including extracellular ATP (Sofoluwe et al., 2019) and high-mobility group box 1 (HMGB1) (Zhang et al., 2022b), signal endogenous damage and are thought to trigger NET formation. PAMPs, such as lipopolysaccharide (LPS) (Dinallo et al., 2019), are external pathogens that can provoke NETosis in UC. Pro-inflammatory cytokines like TNF (Neuenfeldt et al., 2022; Dinallo et al., 2019), and interleukin (IL)-6 (Joshi et al., 2013) contribute to the inflammatory environment and encourage NET formation in UC. C-X-C family chemokines bind to corresponding receptors on neutrophils, causing changes in calcium ion concentration and inducing activation of downstream NOX2 pathways and ERK pathways, promoting the colon residence of neutrophils and the formation of ROS, thereby triggering NETosis (Zhu et al., 2021). A study explored the interaction between inflamed intestinal epithelial cells (IECs) and neutrophils. When IECs were stimulated with LPS, TNF-α, IL-1β, and interferon (IFN)-γ, they transferred LINC00668 via exosomes to neutrophils. This transfer enabled NE to translocate into the nucleus, promoting NET formation (Zhang et al., 2023b). However, in Crohn’s disease (CD), several studies have reported conflicting results. A study using immunohistochemical analysis found that the expression of NET markers NE, MPO, and citH3 in the affected areas of colon tissue of CD patients was significantly higher than that in the control group and non-involved areas, and the expression in the affected areas increased synchronously with the histopathological score (Schroder et al., 2022). However, Dinallo et al. found that elevated TNF-α levels in the colon, PAD4 or NET formation does not increase (Dinallo et al., 2019).

In UC, the timely clearance of NETs is compromised. DNases trigger NET disassembly, followed by macrophage-mediated uptake and degradation (Lazzaretto and Fadeel, 2019). Studies reported diminished NET degradation in UC patients and dextran sulfate sodium (DSS)-induced animal model due to markedly lower DNase I levels (Malíčková et al., 2011; Vrablicova et al., 2020). However, supplementing DNase I only partially restores this function, suggesting other inhibitors of NET breakdown exist (Li et al., 2020). Since anti-NET antibodies can block DNase I access (Hakkim et al., 2010), antineutrophil cytoplasmic antibodies (ANCAs) in UC may act as such protective factors.

### 3.2 Pathophysiological mechanisms of neutrophil extracellular traps

**Figure 2:**
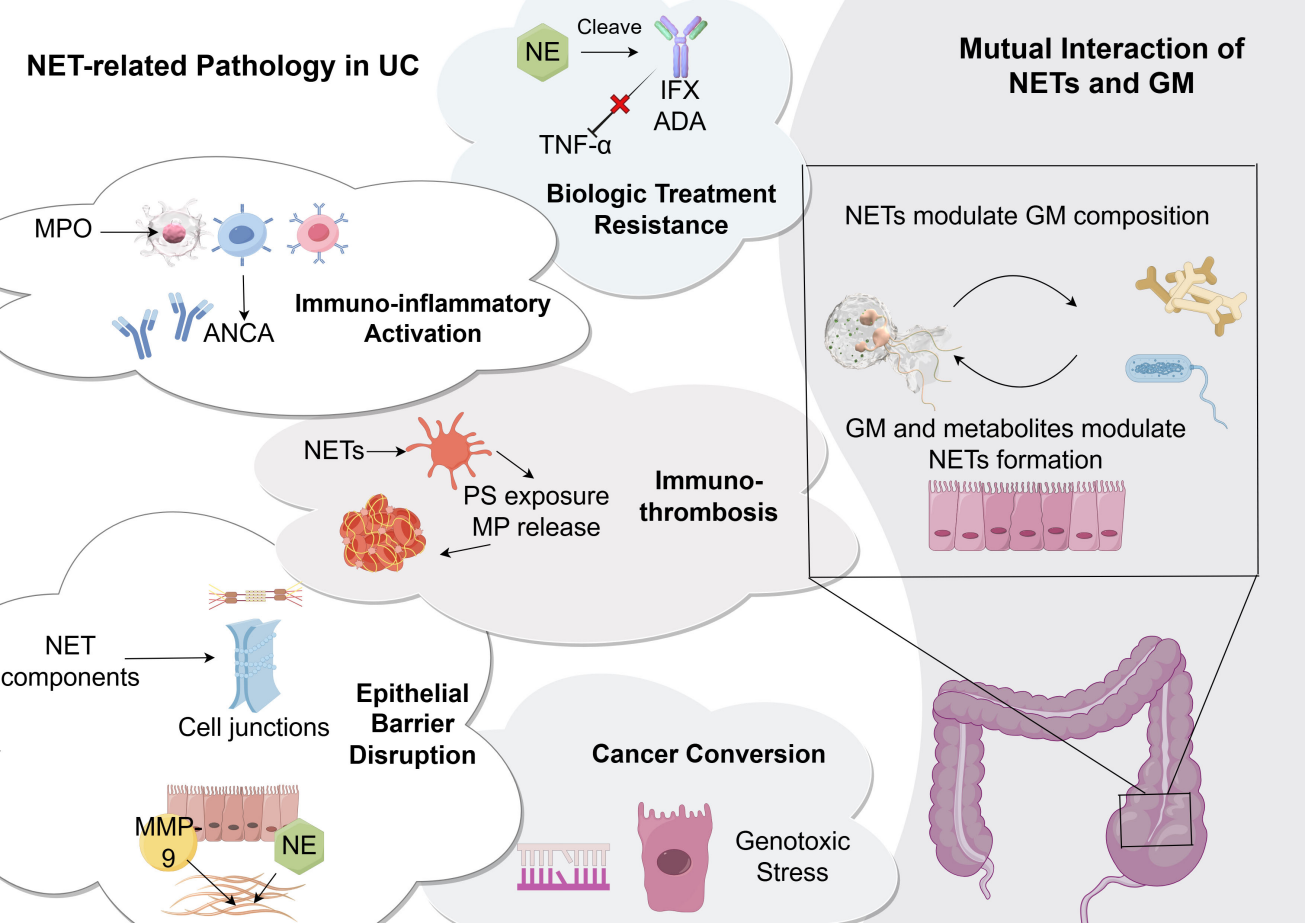
Excessive NETs induce multiple pathological changes and mutually interact with GM. The excessive accumulation of NETs lead to a series of pathological changes in UC, such as aggravating intestinal immuno-inflammatory responses, disrupting epithelial barrier and ECM, activating coagulation cascades, mediating resistance to biological treatment, and inducing malignant transformation. Crucially, NETs mutually interact with GM. NETs modulate GM composition while GM shifts and metabolites modulate NET formation. NET, neutrophil extracellular trap; UC, ulcerative colitis; GM, gut microbiota; TNF, tumor necrosis factor; NE, neutrophil elastase; MMP, matrix metalloproteinases; ANCA, antineutrophil cytoplasmic antibodies; IFX, infliximab; ADA, adalimumab; PS, phosphatidylserine; MP, microparticles.

The excessiveness of NETs triggers multiple pathological effects, including the sustained amplification of immunological and inflammatory signals, and disruption of the intestinal epithelial barrier (See **Figure 2**). These changes actively promote UC development and significantly complicate its treatment.

#### 3.2.1 Immuno-inflammatory responses

Emerging research highlights NETs as pivotal drivers of persistent immune activation and central mediators in UC pathogenesis. Beyond cytokine-induced formation, NETs exacerbate inflammation through further cytokine release, creating a self-perpetuating cycle (Dinallo et al., 2019; Li et al., 2020). They amplify neutrophil activation by triggering the secretion of inflammatory mediators, such as ROS generation via NOX2-dependent mechanisms (Dömer et al., 2021). They also promote C-X-C motif ligand 8 (CXCL8) release, a key chemokine that binds CXCR1/CXCR2 receptors to recruit additional neutrophils (Dömer et al., 2021). Notably, UC-associated NETs carry bioactive IL-1β, a signature feature of colonic inflammation (Angelidou et al., 2018). Exposure to UC-derived NETs upregulated cytokine expression in lamina propria mononuclear cells (LPMCs) and peripheral blood mononuclear cells (PBMCs) (Dinallo et al., 2019). Mechanistically, NETs boost TNF-α and IL-1β secretion in PBMCs through ERK/MAPK signaling and heighten macrophage sensitivity to LPS (Li et al., 2020). Additionally, NETs activate caspase-1 and caspase-8 pathways in J774 macrophages, driving IL-1β production (Hu et al., 2017).

T helper cell 17 (Th17) play a well-established pathogenic role in UC. NET-mediated inflammation initiates robust T cell activation, as NET-deficient mice fail to exhibit inflammatory signal-induced immune cell population changes (Warnatsch et al., 2015). Th17 cell differentiation typically requires an IL-6 and transforming growth factor (TGF)-β-rich milieu, with IL-23 and IL-1β further enhancing their pathogenicity (Jiang et al., 2023). NET components facilitate this process through multiple mechanisms. First, NETs create a pro-inflammatory environment by stimulating myeloid cells to secrete IL-6 and IL-1β (Lambert et al., 2019). Second, histones directly bind to T cell surface toll-like receptor (TLR)-2, triggering STAT3 phosphorylation and RORγt expression independent of cytokines (Wilson et al., 2022). Meanwhile, Th17-derived granulocyte-macrophage colony stimulating factor and IL-17A stimulate neutrophil activation through CXCR1 signaling, establishing a positive feedback loop (Wu et al., 2020). Furthermore, Th17 cells directly trigger NET release (Tohme et al., 2019), leading to excessive NET accumulation and amplifying inflammation.

Key components of NETs such as MPO have been recognized as important autoantigens that stimulate ANCAs, which may function as an indicator of disease progression with extended circulation time (Wen et al., 2022).

#### 3.2.2 Epithelial barrier dysfunction

The intestinal epithelial barrier is vital for defending against external pathogens and ensuring selective permeability of the intestinal mucosa. Its structural integrity is critical for sustaining intestinal homeostasis. Research using DSS-induced colitis models has demonstrated that NETs disrupt intercellular junctions, elevating the expression of E-cadherin, ZO-1 and occludin (Lin et al., 2020). Evidence suggests that NET-derived proteins contribute to mucosal injury. For instance, histones impair intestinal epithelial permeability by disrupting tight junctions and triggering epithelial cell death (Lai et al., 2023). Similarly, cathepsin G cleaves protease-activated receptor 4 (PAR-4), increasing paracellular permeability and exacerbating barrier impairment (Kriaa et al., 2020). Furthermore, NE, cathepsin G, and MMPs degrade extracellular matrix (ECM) components, which has dual consequences. First, fragmented ECM proteins may act as immunogenic stimuli, amplifying inflammatory cell recruitment (Kriaa et al., 2020). Second, since the ECM supports the subepithelial layer, its breakdown compromises the epithelial cell microenvironment, promoting apoptosis. Notably, MMP-9 deficiency mitigates DSS-induced colitis severity, reduces intestinal NET formation, and improves barrier function (Castaneda et al., 2005).

#### 3.2.3 Thromboembolic risks

Growing evidence indicates UC patients demonstrate elevated thrombotic risk (Zezos et al., 2014), with venous thrombosis occurrence rates two or three times greater than healthy individuals (Carvalho et al., 2022). Studies increasingly implicate NETs as pivotal mediators in thrombus formation (Laridan et al., 2019). While NETs normally function in host defense by trapping pathogens through coagulation mechanisms (Massberg et al., 2010), their overactivation may trigger pathological clotting (Pfeiler et al., 2017). NETs promote thrombosis by offering a framework for platelet aggregation and erythrocyte binding via adhesive proteins (Fuchs et al., 2010), and directly engaging with coagulation factor XII (Von Brühl et al., 2012).

In DSS-induced colitis models, NET-mediated thrombosis exhibits paradoxical effects. Neutrophils drive secondary immunothrombosis through PAD4-regulated NET generation, with inadequate formation potentially exacerbating rectal hemorrhage in UC (Leppkes et al., 2022). However, excessive NET production significantly elevates thrombotic risks (Zhang et al., 2023b). Clinical observations identify two key prothrombotic markers in UC patients: enhanced phosphatidylserine (PS) expression on platelets and increased circulating platelet-derived microparticles (He et al., 2016). Mechanistically, NETs stimulate TLR2/4 on platelets, triggering PS surface exposure and microparticle release that collectively promote a hypercoagulable state (Zhang et al., 2023b).

#### 3.2.4 Resistance to biologic treatment

A substantial subset of patients fails to respond to biologic agents (Ben-Horin et al., 2014). As indicated previously, the chronic presence of neutrophils in the colonic mucosa serves as an established indicator of unfavorable therapeutic outcomes to biologic agents. This resistance may stem from the proteolytic microenvironment generated by NET components. Crucially, IFX and adalimumab (ADA), both IgG1 antibodies, contain a vulnerable threonine-histidine bond in their hinge regions. Given the elevated NE activity in UC intestinal mucosa, NE cleaves these therapeutics into Fc monomers and IgG1 fragments. This degradation impairs TNF-α neutralization capacity, directly contributing to anti-TNF non-responsiveness (Curciarello et al., 2020). Notably, cell and organoid studies demonstrate that the NE inhibitor elafin prevents antibody fragmentation, and restores TNF-α blockade efficacy (Curciarello et al., 2020).

#### 3.2.5 Inflammation-mediated carcinogenesis

Neutrophils and NETs significantly influence both inflammatory diseases and cancer. NETs promote tumors by sustaining inflammation and causing DNA damage (Adrover et al., 2023). Chronic inflammation increases genetic mutations, driving abnormal cell growth. Zebrafish studies revealed that injury-induced inflammation enhances pre-neoplastic cell expansion in a neutrophil-dependent manner (Antonio et al., 2015). Particularly, NETs impair tissue repair and sustain chronic injury, creating a pro-tumorigenic niche (Wong et al., 2015).

The link between UC and colorectal cancer highlights NETs as a potential therapeutic target (Itzkowitz and Yio, 2004). UC-driven chronic inflammation generates oxidative stress, genomic instability, and cancer risk. Preclinical studies in colitis-associated cancer models show that PAD4 inhibition (e.g., Cl-amidine) (Wang et al., 2023b) and DNase I-mediated NET degradation (Zhang et al., 2023b) not only alleviate UC pathology but also suppress tumorigenesis. NETs also exacerbate cancer progression and metastasis. In colorectal cancer, NET components enhance malignant cell adhesion, motility, and invasiveness (Khan et al., 2021). Proteolytic enzymes such as NE and MMP9 degrade ECM, reactivating dormant tumor cells and accelerating metastasis (Albrengues et al., 2018).

### 3.3 Summary and perspectives

NETs play a multifaceted and central role in the pathogenesis of UC. An imbalance between enhanced NET formation and impaired clearance leads to their pathological accumulation. These excessive NETs contribute significantly to disease progression through several key mechanisms: they perpetuate immuno-inflammatory activation, disrupt the intestinal epithelial barrier, increase thromboembolic risk, mediate resistance to biologic therapies, and promote inflammation-associated carcinogenesis.

Focusing on NETs may offer a more refined therapeutic approach than merely depleting neutrophils in UC treatment. In cases where NET formation occurs without neutrophil death, neutrophils continue to function in immune defense even after releasing NETs. Future research should focus on elucidating the precise molecular triggers and dynamics of NET formation in UC, understanding the heterogeneity of neutrophil subsets, and developing targeted delivery systems to avoid systemic immunosuppression.

## 4 Interaction of neutrophil extracellular traps and gut microbiota in ulcerative colitis

### 4.1 Gut microbiota in ulcerative colitis

The GM constitutes a complex and dynamic microbial community within the human gastrointestinal tract, engaging in a symbiotic relationship with the host. Dysbiosis, an imbalance between beneficial and pathogenic intestinal bacteria, has been increasingly implicated in host pathophysiology (Quaglio et al., 2022). Current research demonstrates that UC is strongly associated with significant alterations in GM ecology. Large-scale multi-omics studies have confirmed the loss of microbial diversity and disruptions in metabolic activity in UC patients (Lloyd-Price et al., 2019). The pathogenesis of UC-related intestinal inflammation involves a dual microbial mechanism: pathogenic expansion of pro-inflammatory bacterial taxa coupled with depletion of immunomodulatory species. At the phylum level, a characteristic shift in GM composition is observed in UC patients marked by diminished Firmicutes and Bacteroidetes populations and increased Proteobacteria (Walujkar et al., 2014). This shift is further evidenced at the genus level by marked decreases in key butyrate producers, *Roseburia hominis* and *Faecalibacterium prausnitzii*, whose abundance shows significant inverse correlation with clinical disease activity scores and diminished fecal short-chain fatty acid (SCFA) concentrations (Bajer et al., 2017; Machiels et al., 2014). Moreover, the enrichment of pathogenic strains, including adherent-invasive *Escherichia coli* (AIEC) and *enterotoxigenic Bacteroides fragilis*, exacerbates mucosal inflammation through the sustained release of pro-inflammatory mediators and carcinogenic metabolites. This microbial-driven inflammatory cascade not only perpetuates UC progression but also heightens the risk of colorectal carcinogenesis, particularly in the context of chronic inflammation-dysplasia-malignancy transition (Quaglio et al., 2022). In addition, host genetic risk variants for UC may partially mediate disease susceptibility through their effects on the GM, as evidenced by the associations between NOD2 variants and specific bacterial taxa, such as *Faecalibacterium prausnitzii* (Aschard et al., 2019). Another genetic variant, CARD9, influences GM composition and function, leading to impaired tryptophan metabolism and reduced aryl hydrocarbon receptor ligand production, thereby exacerbating intestinal inflammation in DSS-induced colitis (Lamas et al., 2016). Collectively, these findings demonstrate that UC-associated GM dysbiosis fosters a pro-inflammatory microenvironment, contributing to disease pathogenesis and progress.

### 4.2 Mechanism of neutrophil extracellular traps-gut microbiota interaction

Neutrophils engage in complex and intricate interactions with the GM through various pathways. On one hand, they sense microbial-derived components via TLRs and inflammasome signaling pathways, or respond to metabolites through histone deacetylases (HDACs) and G-protein coupled receptors. On the other hand, once recruited to the inflamed colon, neutrophils defend against pathogens by releasing NETs. These interactions contribute to the dual role of NETs in UC, promoting tissue damage while simultaneously limiting microbiota-induced immune responses.

#### 4.2.1 Neutrophil extracellular traps modulate gut microbiota

As primary sentinels of microbial invasion, neutrophils execute essential immunosurveillance functions through sophisticated phagocytic mechanisms. Contemporary research reveals that under inflammatory conditions, neutrophils orchestrate specialized luminal containment structures that selectively encapsulate commensal microbiota (Molloy et al., 2013). Upon exposure to certain pathogenic microbes, neutrophils generate NETs and anucleated cytoplasts which crawl and engulf bacteria (Yipp et al., 2012). The unique structure of NETs enables them to capture, neutralize, eliminate pathogens and prevent their dissemination (Papayannopoulos, 2018).

CD177^+^ neutrophils are characterized by high ROS production and the formation of NETs. An increased expression of CD177^+^ neutrophils has been observed in the peripheral blood and colon of UC patients. However, CD177-/- mice exhibited more severe colitis. Sequencing of CD177^+^ and CD177^-^ neutrophils from UC patients revealed that the high expression of CD177^+^ neutrophils is associated with genes related to antimicrobial responses and ROS formation, suggesting that NETs in colitis may limit immune activation by combating intestinal bacterial translocation (Zhou et al., 2018).

Another study investigated the role of neutrophils in colonic inflammation using C57BL/6 and C3H/HeN mice (Sanchez-Garrido et al., 2024). The *Citrobacter rodentium*-induced colitis model shows certain similarities to human UC, particularly in the C3H/HeN strain. Following *Citrobacter rodentium* intervention, normal neutrophil activation and NET formation were observed in C57BL/6 mice, promoting pathogen clearance. Subsequently, neutrophils were either phagocytized or underwent reverse migration, leading to the resolution of inflammation. In contrast, C3H/HeN mice exhibited defects in neutrophil activation and migration, with numerous neutrophils being trapped in the submucosa, where they underwent harmful NETosis without direct bacterial contact. This resulted in the release of NE and MPO, leading to tissue damage and more severe colitis, even death. Additionally, a decrease in the expression of CD11b and CXCR4 on neutrophils was observed in both UC patients and C3H/HeN colitis mice, which could impair neutrophil activation and reverse migration.

While the effects of NETs on pathogens may offer some beneficial roles, in UC, the negative impact of the NETs-GM imbalance should be acknowledged. In UC, persistent inflammation and microbial imbalance contribute to the degradation of the protective mucus layer and compromise the structural integrity of the gut epithelial barrier. This breakdown facilitates heightened interactions between GM and epithelial cells, as well as with neutrophils that migrate to the lamina propria, exacerbating inflammatory responses and enhancing NET-microbiota interactions (Danne et al., 2024). Furthermore, *Clostridium difficile* is a common concomitant infection during acute flare-ups of UC that can exacerbate intestinal inflammation and lead to poor prognosis. A study demonstrated that short-term colonization of *Clostridium difficile* in mice with DSS-induced colitis significantly altered the microbiota profile, mainly characterized by a reduction in the abundance of *g_Prevotellaceae_UCG-001* and *g_Muribaculaceae* (Dong et al., 2023). This resulted in robust neutrophil infiltration and the generation of NETs. Subsequent inhibition of CXCR2 activity markedly suppressed the activation of neutrophils in the gut and led to an improvement in histological inflammation. Intriguingly, our previous research has implicated CXCR2 as a UC susceptibility locus, with mechanistic studies linking its activity to neutrophil chemotaxis and PAD4-dependent NETosis (Xv et al., 2024), and CXCR2 deficiency was demonstrated to induce marked microbial community shifts (Jee et al., 2017).

These findings collectively delineate an intricate immunoregulatory axis wherein neutrophils and NETs undergo transcriptional reprogramming that shapes gut ecology, and engage in bidirectional crosstalk with commensal species. While indispensable for mucosal homeostasis, their antimicrobial effector functions demonstrate context-dependent outcomes, which is protective during steady-state conditions yet potentially deleterious when dysregulated in chronic inflammation.

#### 4.2.2 Gut microbiota regulate neutrophil extracellular trap formation

Emerging evidence demonstrates that the GM serves as a pivotal regulator of neutrophil biology, governing their production, activation, and functional maturation through intricate mechanisms. The GM regulates neutrophil production through both direct and indirect pathways. Indirect modulation occurs via interactions between intestinal microbes and epithelial cells, innate lymphoid cell, or stromal cells. Alternatively, direct control is mediated through the gut-bone marrow signaling axis (Danne et al., 2024). Notably, GM induces a hyperactivated neutrophil subset characterized by upregulated αMβ2 integrin expression, enhanced adhesion molecule activation, and increased NET formation capacity, which polarizes neutrophils toward a pro-inflammatory phenotype in pathological conditions (Zhang et al., 2015). The GM suppresses neutrophil hyperreactivity and NETosis in mesenteric ischemia-reperfusion injury via TLR4/TRIF signaling, while promoting immunovigilance through enhanced neutrophil recruitment, as evidenced by gnotobiotic mouse models showing elevated NET formation, which was reversed by LPS desensitization or TRIF deficiency (Ascher et al., 2020). Notably, the microbiota exerts context-dependent control over NETosis. LPS from *Pseudomonas aeruginosa* and certain *E. coli* species trigger “suicidal” NETosis dependent on autophagy/ROS in isolated neutrophils, while promoting “vital” NETosis mediated by TLR4/CD62P in whole blood, demonstrating how diverse microbial-origin of LPS collectively regulate NET formation (Pieterse et al., 2016). GM dysregulation disrupts neutrophil homeostasis, as evidenced by antibiotic-induced dysbiosis synergizing with AIEC infection to exacerbate NETosis and oxidative damage (Vong et al., 2016). Meanwhile, NET release is correlated with their age in circulation, and neutrophil ageing is driven by the microbiota via TLRs and myeloid differentiation factor 88-mediated signalling pathways (Zhang et al., 2015).

Emerging evidence highlights the pivotal role of the microbial metabolites in both preserving intestinal equilibrium, orchestrating immune system regulation, and contributing to UC development. SCFAs, organic compounds containing fewer than six carbon atoms, are generated through bacterial breakdown of non-digestible carbohydrates in the gut. Butyrate is capable of attenuating intestinal inflammation in experimental models by modulating neutrophil-mediated immune pathways, including suppression of inflammatory cytokine production and NET release (Li et al., 2021b). Butyrate modulates neutrophil activity in IBD through multiple mechanisms. Experimental studies demonstrate that dietary supplementation of butyrate attenuates DSS-induced colonic inflammation, primarily through HDAC-mediated suppression of NET formation (Li et al., 2021b). Clinical investigations further reveal that butyrate treatment significantly reduces ROS generation and subsequent NET release in neutrophils isolated from IBD patients (Li et al., 2021b). Interestingly, another study showed that physiological concentrations of GM-derived SCFAs, particularly butyrate, acetate, and propionate, induce NET formation through FFA2R/Gαq11/NADPH oxidase signaling (Íñiguez-Gutiérrez et al., 2020).

### 4.3 Summary and perspectives

**Figure 3:**
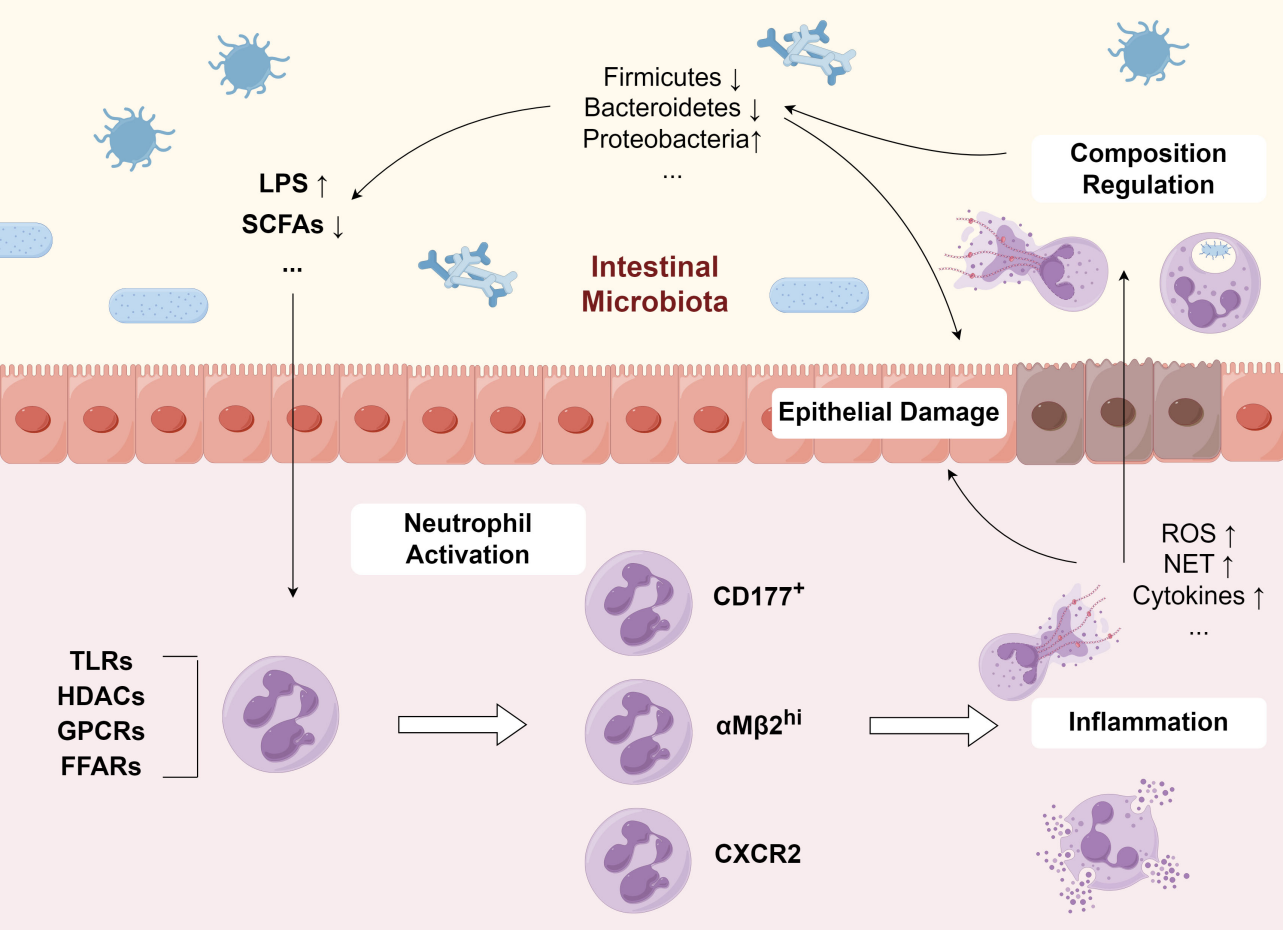
Reciprocal interplay between GM and neutrophil activation. GM and its metabolites stimulate the activation of diverse neutrophil subsets, enhancing their production of ROS and NETs, which promotes inflammation. In turn, the released NETs modulate the composition and function of the GM, thereby creating a vicious cycle that exacerbates disease progression. GM, gut microbiota; NET, neutrophil extracellular traps; LPS, lipopolysaccharide; SCFA, short-chain fatty acid; TLR, toll-like receptor; HDAC, histone deacetylase; GPCR, G protein-coupled receptor; FFAR, free fatty acid receptor; CXCR, C-X-C motif chemokine receptor; ROS, reactive oxygen species.

The interplay between NETs and GM in UC is not unidirectional but constitutes a dynamic feedback loop that amplifies and perpetuates intestinal inflammation (**Figure 3**). On one hand, dysbiotic microbiota and their metabolites modulate neutrophil recruitment, activation, and NETosis via pattern recognition receptors and metabolite-sensing pathways. On the other hand, NETs directly shape microbial community structure through antimicrobial components and physical trapping, further influencing epithelial barrier integrity and mucosal immune responses. This reciprocal interaction creates a self-sustaining inflammatory cycle: dysbiosis promotes aberrant NET formation, which in turn exacerbates microbial imbalance, barrier disruption, and chronic inflammation. Elucidating this feedback loop is critical for understanding UC pathogenesis and designing interventions. Considering the pivotal role of neutrophils in innate immunity, indiscriminate depletion of neutrophils may not be beneficial for colonic inflammation. The "double-edged sword" effect of NETs necessitates more precise regulation. Targeting neutrophils through the GM offers a potential strategy to regulate the balance between NETs' pro-inflammatory effects and their antimicrobial protection.

## 5 Traditional Chinese medicine targeting the neutrophil extracellular traps-gut microbiota axis

Therapeutically, targeting neutrophils is a double-edged sword. Inhibiting or depleting these cells carries risks including increased susceptibility to severe infections due to neutropenia. Current neutrophil-targeted therapies have largely been disappointing in clinical settings (Danne et al., 2024). Instead of inhibiting or depleting these cells, functional modulation may offer a more effective approach to disease management. Due to the microbiome-host interaction, modulating the intestinal microbiota to influence neutrophil activity, particularly in the context of UC, might be a promising strategy.

Research highlights the therapeutic promise of TCM in managing UC. In randomized controlled trials (RCTs), medicine like indigo naturalis (Naganuma et al., 2018), Fufangkushen colon-coated capsule (Gong et al., 2012), Qing-Chang-Hua-Shi granules (Shen et al., 2021), and modified Wumei pills (Li et al., 2025a) exhibit superior efficacy in UC compared with placebo or western medicine alone. Studies demonstrate that various herbal compounds effectively treat UC through modulating multiple cellular signaling pathways (Zhang et al., 2022a). Evidence further suggests specific TCM-derived therapies can influence NETs and GM composition, mitigating colonic inflammation and associated risks like thrombosis and malignancy. This multi-targeted approach underscores significant potential for clinical application.

### 5.1 Single herbal extracts targeting neutrophil extracellular traps

Several natural compounds have been demonstrated to directly inhibit NET formation in the models of UC, and the main targets highly concentrated on PAD4, NE, MPO, and related signaling pathways. Berberine effectively improved the clinical efficacy, reduced inflammation, and alleviated symptoms in UC patients (Li et al., 2025a) and DSS-induced models (Deng et al., 2020), while reducing NET formation, a key mechanism underlying its anti-inflammatory and anti-thrombotic effects in UC. It achieves this by suppressing nuclear translocation of NE, mediated through disrupting the interaction between exosome-transferred lncRNA LINC00668 and NE (Zhang et al., 2023b). Berbamine reduces PAD4 expression and NET markers (cit-H3, NE, MPO) in neutrophils and colonic tissues of DSS-mice (Tang et al., 2024). Ferulic acid suppresses neutrophil migration and NET generation within the colons of mice with UC. Crucially, its anti-inflammatory effect was absent in mice lacking neutrophils, demonstrating that neutrophil activity is essential for ferulic acid's mitigation of colitis (Han et al., 2023). Forsythiaside A treatment improved DSS-induced colitis, reduced PAD4-associated NET formation in colon tissue. Similar results were achieved in cultured neutrophils, where forsythiaside A pretreatment also suppressed PAD4 expression and NETosis induced by PMA (Wang et al., 2025). Arbutin significantly lessens inflammatory factors, neutrophil infiltration, and NET formation in UC models by inhibiting the Erk pathway in neutrophils (Qin et al., 2024). Dihydromyricetin also alleviates colon inflammation, reduces proinflammatory cytokines, improves intestinal epithelium integrity, and inhibits NETs *(* Ma et al., 2024). *In vitro* studies suggest it repairs the mucosal barrier by targeting NETs, primarily via inhibiting the HIF-1α/VEGFA signaling pathway, which is a key regulator of hypoxia-driven neutrophil recruitment and activation in inflamed intestinal tissue.

In addition, several compounds effective in UC may have the anti-NET effects not limited to the UC model. These effects have also been confirmed in models of sepsis, arthritis, viral infections, chemical injury, or cancer metastasis. This cross-model NET inhibitory activity provides clues for further investigations into the anti-colitis mechanisms.

Glycosides, including ginsenosides and forsynthiaside B, are demonstrated to alleviate DSS-induced colitis (Hwang et al., 2017; Cheng et al., 2022). Forsynthiaside B downregulates PAD4 expression and NET formation in a sepsis model (He et al., 2022). Ginsenoside Rg1 suppresses NET generation in pulmonary tissues and counteracts their tumor-promoting effects (Lu et al., 2024), while Rg5 alleviated experimental deep vein thrombosis by inhibiting NETosis and neutrophil-driven inflammation through P2RY12 signaling (Chen et al., 2022).

Flavonoids that have efficacy in UC include baicalin and luteolin. Baicalin reduced the level of MDA, IL-1β, TNF-α and MPO in the colon of UC model (Shen et al., 2019) and inhibited NET formation and neutrophil chemotaxis in viral infection models (Li et al., 2023). Luteolin alleviates UC symptoms and inflammatory responses in animal models (Liu et al., 2020). Further mechanistic studies revealed its suppression of ROS production and NET formation in human neutrophils, mediated through the Raf1-MEK1-ERK signaling cascade (Yang et al., 2018).

In terms of phenolic compounds, curcumin ameliorates symptoms in mild-to-moderate UC patients (Sadeghi et al., 2020; Lang et al., 2015), induces responses and remission (Ben-Horin et al., 2024), and reduces recurrence (Hanai et al., 2006), with parallel experimental evidence demonstrating its suppression of polymorphonuclear neutrophil chemotaxis (Larmonier et al., 2011) and MPO activity (Liu et al., 2013) in IBD models. Further mechanistic research revealed that curcumin attenuates polybrominated diphenyl ether-induced neutrophil ROS generation and NET release by activating the Nrf2 pathway (Ye et al., 2021).Total phenolic acid extract and tanshinone extract from *Salvia miltiorrhiza* effectively ameliorate DSS-induced colitis (Peng et al., 2021). Among them, tanshinone IIA suppressed neutrophil infiltration and NET formation in rheumatoid arthritis models (Zhang et al., 2017). Dihydrotanshinone I reversed PMA-triggered NET formation in 4T1 breast cancer cells by scavenging ROS, and reducing Ly6G^+^MPO^+^ neutrophil accumulation and citH3 expression in lung tissue, thereby inhibiting NET-driven metastatic dissemination (Zhao et al., 2022). Paeonol has been demonstrated to mitigate TNBS-induced colitis (Zong et al., 2017). APPA (containing paeonol), reduced neutrophil degranulation, ROS, and NETs without compromising host defense (Cross et al., 2020).

As for terpenoids and polyssacharides, andrographolide derivatives mitigate DSS-induced UC by suppressing NF-κB and MAPK signaling cascades (Gao et al., 2018), concurrently attenuating polymorphonuclear leukocyte infiltration and NET formation in arthritis models (Li et al., 2019). Similarly, carnosic acid alleviates acute DSS-triggered colonic inflammation through reduced MPO activity (Yang et al., 2017), while directly restraining neutrophil activation via diminished superoxide anion/ROS biosynthesis, elastase secretion, and cellular adhesion. Mechanistically, it blocks NETosis by inhibiting Erk/JNK phosphorylation (Tsai et al., 2023). Ganoderma atrum-derived water-soluble PSG-1 restores intestinal physical and immune barriers in murine colitis (Zheng et al., 2020), whereas Ganoderma lucidum peptide-polysaccharide GL-PPSQ2 counters intestinal ischemia-reperfusion injury by preserving mucosal integrity, enhancing tight junctions, reducing inflammation, and suppressing MPO/citH3 expression linked to NET-associated pathology (Lin et al., 2023).

NETs appear to be a key "bridge" connecting inflammation, tissue damage, thrombosis, and other complications, making itself a significant target of UC treatment. Most effective compounds exhibit pleiotropic effects, including NET inhibition, anti-inflammation, antioxidation, and barrier repair.

### 5.2 Single herbal extracts targeting gut microbiota

Many core compounds mentioned above possess dual capabilities of regulating GM and inhibiting neutrophil activation/NET formation (See **Table 1**). On one hand, they regulate the GM to reduce pro-inflammatory signals and antigen stimulation, indirectly inhibiting excessive immune responses; on the other hand, they directly inhibit key pathogenic effects of NETs. This intervention targeting the "microbiome-immune" axis in a coordinated manner could be the key to their superior efficacy compared to single-target interventions.

**Table 1:** Single herb extracts and compounds targeting NETs and GM in UC.

Active compound	Chemical structure	Anti-NET mechanisms	Gut microbiota effects	Key benefits in UC	References
Arbutin	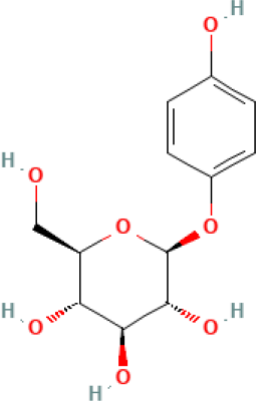	Inhibits Erk pathway; Suppresses neutrophil infiltration and NET formation	↓ *Mucispirillum* *Schaedleri, Clostridium perfringens*; ↑ *Bacteroides acidifaciens, Parabacteroides* *Disasonis, Clostridium cocleatum, Oscillospira*, Lachnospiraceae	Reduction of inflammation and neutrophil infiltration	(Qin et al., 2024)
Berberine	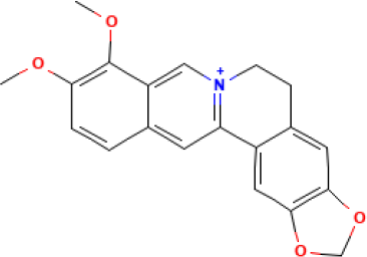	Inhibits NE nuclear translocation; disrupts interaction of LINC00668/NE	↑ *Lactobacillus/Lactococcus*; ↓ *Bacteroides, segmented filamentous bacteria*, Enterobacteriaceae	Anti-inflammation, anti-thrombosis, barrier repair, immune homeostasis regulation	(Zhang et al., 2023b; Dong et al., 2022)
Curcumin	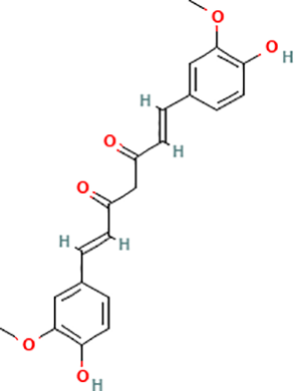	Activates Nrf2; Inhibits polymorphonuclear neutrophil chemokinesis, MPO activity, ROS, NETs	↑ *Akkermansia, Roseburia, F16, Coprococcus*; ↓ Enterococcaceae, Aerococcaceae, *Turicibacter*	Anti-inflammation, antioxidation	(Larmonier et al., 2011; Liu et al., 2013; Ye et al., 2021; Guo et al., 2022)
Dihydromyricetin	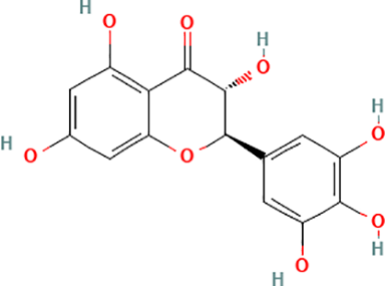	Inhibits HIF-1α/VEGFA pathway and neutrophil recruitment	↑ *Lactobacillus, Akkermansia*	Barrier repair, anti-inflammation, bile acid metabolism restoration	(Ma et al., 2024; Dong et al., 2021)
Forsythiaside A	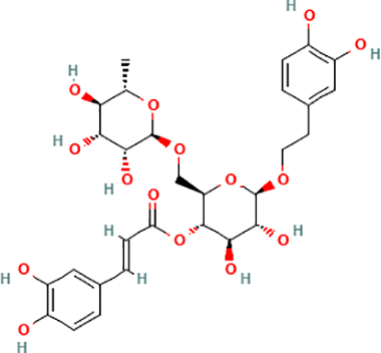	Suppresses PAD4/NETosis	↑ Simpson Index; ↓Oscillospiraceae, Bacteroides, Colidextribacter*;* ↑ acetic acid, valeric acid, isovaleric acid, propanoic acid	Anti-inflammation, tight junction increase	(Wang et al., 2025)
Ginsenoside Rg1	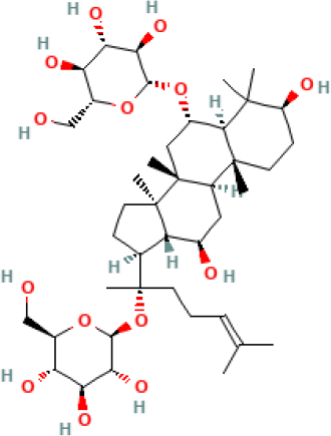	Suppresses NETs in tumors	↑ Sobs, Ace, Chao indexes; ↓ *Romboutsia*; ↑ *Rikenellaceae_RC9_gut_group, Lachnospiraceae_NK4A136_group, Enterorhabdus, Desulfovibrio, Alistipes*	Anti-inflammation, lipid metabolism regulation, Th1/Th2/Th17 cell differentiation regulation	(Lu et al., 2024; Zhong et al., 2023)
Luteolin	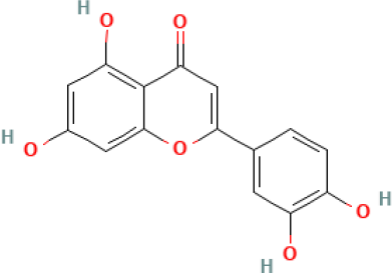	Inhibits Raf1-MEK1-ERK, superoxide anion generation, ROS, NETs	↓ *Lactobacillus, Prevotella_9*	Cytokine reduction, anti-oxidation, barrier Repair	(Yang et al., 2018; Li et al., 2021a)
Paeonol	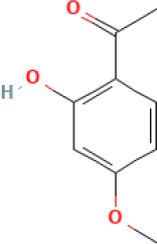	Reduces neutrophil degranulation, ROS, and NETs	↑ SCFAs; ↑ C. butyricum	Anti-inflammation, barrier restoration	(Cross et al., 2020; Zhao et al., 2023)

Some compounds provide the strong causal evidence with microbiome and treatment. Through antibiotic-induced microbiome depletion or fecal microbiota transplantation (FMT) experiments, it has been demonstrated that their anti-UC efficacy completely relies on the presence and regulation of the GM. Berberine increases the relative abundance of beneficial bacteria compared to the model group, including up-regulating *Lactobacillus/Lactococcus*. On the other hand, harmful bacteria such as *Bacteroides*, *segmented filamentous bacteria*, and Enterobacteriaceae were reduced. Additionally, depletion of microbiota through antibiotic treatment significantly reversed berberine's therapeutic effects, suggesting that its anti-colitis action is microbiota-dependent (Dong et al., 2022). Dihydromyricetin has also shown the effect of alleviating gut dysbiosis in colitis mice. Antibiotic-mediated microflora depletion and FMT established that its therapeutic efficacy depends on GM. It restored microbial bile acid metabolism during colitis development, and significantly enriched beneficial *Lactobacillus* and *Akkermansia* genera (Dong et al., 2021). Paeonol improved intestinal microecological imbalance, and promoted the production of SCFAs. In particular, C. butyricum was identified as a key bacterium responsible for the intestinal barrier repair effect of paeonol in UC mice (Zhao et al., 2023).

Several other compounds have also shown the ability to significantly modulate the microbiota diversity and composition in UC. Arbutin decreased the abundance of potentially harmful bacteria such as *Shigella* Induced by DSS at the genus level. At the species level, arbutin reversed the increased abundance of pathogenic species, including *Mucispirillum schaedleri* and *Clostridium perfringens*, alongside the decrease in beneficial anti-inflammatory probiotics and butyrate-producing bacteria exhibited in DSS-treated mice (Qin et al., 2024). Forsythiaside A treatment not only increased the expression of the tight junction protein and decreased inflammatory cytokines in the colon, but also alleviate gut dysbiosis in colitis mice (Wang et al., 2025). Luteolin treatment modulated GM structure in UC rats, elevating beneficial genera (*Lactobacillus*, *Bacteroides*, *Roseburia*, *Butyricicoccus*) and suppressing DSS-induced increases in *Prevotella_9* and *Lactobacillus* proportions (Li et al., 2021a). Curcumin has been demonstrated to be beneficial for modulating abundance of some specific bacteria in DSS-induced mice, including *Akkermansia* and *Roseburia*, as well as families such as F16 and Aerococcaceae (Guo et al., 2022). Ginsenoside Rg1 improved the composition of GM in obese mice with colitis, with increases in alpha diversity indexes, a significant down-regulation of *Romboutsia*, and up-regulation of *Enterorhabdus, Desulfovibrio*, and *Alistipes* (Zhong et al., 2023).

Studies on some compounds have revealed broader connections that they can simultaneously inhibit neutrophil recruitment/activation and regulate GM balance. The early prevention effect of ursolic acid could effectively alleviated UC inflammation, reduce neutrophils infiltration, and reverse the reduction of the richness of intestinal flora, meanwhile regulating inflammatory and fatty acid metabolism signaling pathways (Sheng et al., 2021). The nanocrystals of indigo and indirubin exhibited improved therapeutic efficacy in DSS-induced mice via downregulating the expression of macrophages, neutrophils, and dendritic cells and maintaining intestinal flora homeostasis (Xie et al., 2023). Astragalin treatment reduced the expression of pro-inflammatory cytokines, inhibited colonic infiltration by neutrophils, ameliorated metabolic endotoxemia, and partially reversed the alterations in the GM in colitis mice, mainly by increasing the abundance of potentially beneficial bacteria (such as Ruminococcaceae) and decreasing the abundance of potentially harmful bacteria (such as *Escherichia-Shigella*) (Peng et al., 2020).

Meanwhile, many other TCM compounds exhibit potent GM-modulating properties, for which evidence of neutrophil/NETs involvement is still sparse. Nevertheless, their established role in gut health suggests a potential, yet unexplored, impact on neutrophilic inflammation, meriting further investigation. Plantamajoside administration reshaped the GM by elevating the abundance of Bacteroidota and Verrucomicrobiota while reducing Firmicutes and Proteobacteria. At the genus level, it suppressed pathogenic bacteria such as *Turicibacter* and promoted beneficial taxa like *[Eubacterium]_xylanophilum_group* (Jia et al., 2025). Morin also modulated the GM composition in DSS-induced models by promoting beneficial bacteria such as Muribaculaceae and Erysipelotrichaceae, while simultaneously decreasing the abundance of detrimental bacterial groups (Qiu et al., 2024). Pimpinellin increased beneficial gut probiotics (S24-7) while reducing harmful bacteria (Enterobacteriaceae) (Lv et al., 2025). Purslane treatment enhanced the diversity of the GM, elevating the abundance of *Butyricoccus* and *Bifidobacterium*, while reducing the levels of *Bacteroides* and *Parabacteroides*. Serum metabolomics further revealed that the dysregulation of 39 metabolites was substantially ameliorated following purslane administration (Li et al., 2025b). C. pilosula polysaccharide, composed of rhamnose, arabinose, galactose, glucose, and galacturonic acid, notably elevated the Firmicutes/Bacteroidetes ratio and enhanced the proliferation of beneficial bacterial genera, including *g:Ligilactobacillus*, and *g_Akkermansia*. This shift in microbial community further stimulated the production of acetic acid and butyric acid. The rise in SCFAs subsequently mitigated inflammatory responses via the GPR/NLRP3 signaling pathways (Zhou et al., 2025). Ganoderic acid promoted tryptophan metabolism, subsequently activated the aryl hydrocarbon receptor, and triggered the production of IL-22, which was mediated by GM (Kou et al., 2024). Puerarin counteracted the increased abundance of *Akkermansia muciniphila* in DSS mice, and effectively inhibited the activation of M1-like macrophage triggered by the baterial secreted protein Amuc_2172 (Li et al., 2025b). Formononetin also alleviated UC through restoring the balance of M1/M2 macrophage polarization, which was GM-dependent (Xiao et al., 2024).

The regulation of these compounds is bidirectional, which promotes beneficial bacteria, especially SCFA-producing and barrier-related bacteria, and inhibiting pathogenic bacteria, restoring both composition and function of microbiome. Many compounds show the ability to simultaneously regulate GM and neutrophils, suggesting their integrated effect on the "microbiome-immune-barrier" axis.

### 5.3 Formulas targeting the neutrophil extracellular traps-gut microbiota crosstalk

A defining characteristic of TCM in treating complex diseases like UC lies in its holistic approach, typically realized through a multi-component, multi-target, and multi-pathway therapeutic strategy. Significantly, as highlighted in the preceding sections, numerous bioactive TCM-derived components exhibit dual or even multiple therapeutic properties. Specific compounds discussed earlier demonstrate a remarkable capacity to simultaneously modulate NETs and intervene in GM dysbiosis. Building upon this foundation of multi-targeting constituents, this section focuses on the synergistic mechanisms of several representative TCM formulas specifically employed for UC management (See **Table 2**).

**Table 2:** Traditional Chinese medicine formulas treating UC via NETs and microbiota.

TCM formula	Key herbs	NETs inhibition	Gut microbiota modulation	Synergistic mechanisms & benefits	References
Baitouweng Decoction	*Radix pulsatillae, Cortex phellodendri, Rhizoma coptidis, Cortex fraxini*	↓ CXCL1/CXCL2, neutrophil infiltration; ↓ PAD4, NE, MPO, citH3, MMP	↑ Firmicutes, Proteobacteria, Actinobacteria, Tenericutes*, TM7*; ↓ Bacteroidetes	Anti-inflammation; bile acid modulation	(Wang et al., 2024; Hua et al., 2021)
Da-Yuan-Yin Decoction	*Semen Areca, Magnolia officinalis, Fructus tsaoko, Rhizoma Anemarrhee, Paeonia obovata, Radix Scutellariae, Radix Glycyrrhizae*	↓ PADI4, MPO, NE, cit-H3, TLR4	↑ Simpson index; ↑ *Akkermansia*, Firmicutes/Bacteroidetes; ↓ *Klebsiella, Parabacteroides, Escherichia-Shigella*, Colidextribacter, Clostridioides*, Parasutterella*; ↑ SCFAs	Restores tight junctions; anti-inflammation	(Yang et al., 2024, Yang et al., 2025)
Gegen Qinlian Decoction	*Radix Puerariae*, *Scutellaria hypericifolia*, *Rhizoma Coptidis*, *Radix Glycyrrhizae*	↓ Neutrophil recruitment (implied NET suppression)	↑ *Ruminococcaceae_UCG-01*3; ↓ *Parabacteroides, [Eubacterium]_fissicatena_group, Akkermansia*	Anti-inflammation	(Li et al., 2022b)
Huangqin Decoction	*Radix Scutellaria, Radix Paeoniae Alba, Radix Glycyrrhizae, Fructus Zizyphi Jujubae*	↓ PAD4/NETs	↑ ACE, Chao1 indexes; ↑ Firmicutes; ↓ Bacteroidetes	Boosts CD8+ T cell immunosurveillance; reduces UC-associated cancer risk	(Pan et al., 2022; Li et al., 2022a)
Kui-jie-ling Capsule	*Radix Astragali, Radix et Rhizoma Rhei, Retinervus Luffae Fructus, Margarita*	↓ cit-H3, MPO	↓ Bacteroides*, Escherichia–Shigella*; ↑ Muribaculaceae; ↑ acetic acid, propionic acid, butyric acid, acetic acid	Restores intestinal homeostasis	(Li et al., 2024)
Si-Jun-Zi Decoction	*Radix Codonopsis pilosulae, Rhizoma Atractylodis macrocephalae, Poriae Alba, Radix Glycyrrhizae*	↓ TNF-α/NE and IL-1β/NE co-localization in neutrophils	↓ Proteobacteria*, Escherichia-Shigella*; ↑ bile acid biosynthesis	Microbiota-dependent anti-inflammation; enhances barrier integrity	(Wu et al., 2023; Zhang et al., 2023a)

Baitouweng decoction has been demonstrated in RCTs that they can improve the symptoms of colitis and inhibit inflammation (Xie et al., 2024). The extract from Baitouweng decoction showed therapeutic potential in a murine model of UC by reducing chemokine levels (CXCL1 and CXCL2) and inhibiting neutrophil infiltration in the colon. It also downregulated the expression of key proteins associated with NET formation (PAD4, NE, MPO, citH3, MMP) (Wang et al., 2024). The decoction also enhanced GM diversity and the relative abundance of Firmicutes, Proteobacteria, Actinobacteria, among others, while restoring Bacteroidetes levels. Furthermore, it increased farnesoid X receptor and Takeda G protein-coupled receptor 5 expression in the liver, alleviating DSS-induced symptoms through the modulation of bile acids and GM (Hua et al., 2021).

Research on UC-associated colorectal cancer demonstrated that Huangqin decoction delayed carcinogenesis initiation. Beyond this protective effect, it also mitigated inflammation and boosted CD8^+^ T cell immunosurveillance. Mechanistically, these benefits stemmed from NETs downregulation, and linked to PAD4 deactivation (Pan et al., 2022). Moreover, the decoction modulated the DSS-induced gut dysbiosis (Li et al., 2022a).

Si-Jun-Zi decoction significantly alleviated colonic tissue damage, enhanced intestinal barrier integrity, and markedly suppressed the abundance of the phylum Proteobacteria and the genus *Escherichia-Shigella*. The regulation of GM leads to modulation of bile acid biosynthesis. The decoction was further proved to exert anti-inflammatory activities in a GM-dependent manner (Wu et al., 2023). Additional experiments showed that the decoction can reduce the co-localization of TNF-α/NE and IL-1β/NE in PMA-stimulated neutrophils, which exhibits the potential of NET regulation (Zhang et al., 2023a).

Da-Yuan-Yin Decoction protected the intestinal barrier by restoring levels of tight junction proteins. Furthermore, it suppressed the expression of NET-related genes (PADI4, MPO, NE) and TLR4. Correlation analysis indicated that claudin-1 levels inversely correlated with both MPO and PADI4, while TLR4 levels showed a positive correlation with NE (Yang et al., 2024). Another study corroborated these findings that the decoction downregulated cit-H3, PADI4, and MPO expressions in the lung, stomach, and colon in UC mice. Moreover, the intervention restored GM diversity and abundance, while ameliorating metabolic dysregulation by increasing total SCFA content (Yang et al., 2025).

Gegen Qinlian Decoction significantly ameliorated colitis and concurrent pulmonary inflammation, as evidenced by the down-regulated expressions of inflammatory cytokines and the suppressed recruitment of neutrophils. Meanwhile, the decoction greatly improved intestinal microbiota imbalance in the feces of colitis mice (Li et al., 2022b).

Kui-jie-ling capsule, specifically designed and applied in the treatment of UC in China, alleviated UC through multi-pathway mechanisms. One study demonstrated that, when combined with ADA, it inhibits NET-related markers (cit-H3, MPO) and restores intestinal homeostasis, which is superior to ADA alone (Li et al., 2024). The intervention restored the content of acetic acid, propionic acid, and butyric acid in the colon of the DSS mice, while decreasing isobutyric acid, valerate acid, and isovalerate acid. FMT further verified the protective effect of Kui-jie-ling capsule is dependent on microbiota.

The therapeutic superiority of these TCM formulas lies in their ability to concurrently disrupt the vicious cycle between NET formation and GM dysbiosis by simultaneously targeting NET-related inflammatory pathways and restoring microbial balance. This dual perturbation of interconnected pathological axes enables a comprehensive dampening of UC progression and underscores the holistic mechanism through which multi-target TCM achieves synergistic therapeutic outcomes.

### 5.4 Research gaps and future directions

Despite the promising evidence supporting the role of TCM in modulating the NETs-GM axis in UC, several research gaps remain to be addressed. Most existing studies are preclinical, relying heavily on animal models and *in vitro* systems. Clinical validation in well-designed human trials is scarce, limiting the translation of these findings into evidence-based therapies. The precise mechanisms through which TCM components simultaneously regulate NET formation and microbial communities are still not fully elucidated.

A key question is how the multi-target nature of TCM comprehensively disrupts the vicious cycle of inflammation, NET release, dysbiosis, and barrier damage. This includes clarifying how TCM restores immune-microbiome crosstalk not by isolated inhibition, but through system-level reprogramming that resolves neutrophilic inflammation while reestablishing microbial homeostasis and mucosal integrity. Further research should prioritize conducting rigorous RCTss to evaluate the efficacy and safety of TCM compounds and formulas in UC patients, especially in comparison with conventional therapeutics. Researchers should consider utilizing multi-omics approaches to unravel the complex interactions between specific TCM compounds, NETs, and GM, and to identify key microbial taxa and immune pathways involved. Addressing these gaps will not only enhance our understanding of TCM’s holistic regulatory capacity but also facilitate the development of novel integrative treatment strategies for UC and other immune-mediated diseases.

Moreover, further technical precision is needed in research on the mechanisms by which TCM inhibits NETs and microbiota in UC. (1) The characterization of NET formation would benefit from the use of multiple methods, such as multiplex immunohistochemical techniques for co-localization analysis of DNA-MPO/citH3/NE complexes and live-cell imaging of neutrophils using electron microscopy (Boeltz et al., 2019). This is crucial because single markers such as MPO, NE, or cfDNA may not clearly indicate NET formation. (2) Current research in this area occasionally lacks methodological rigor, making it difficult to conclusively establish the targeting effects of TCM on NETs. Future investigations should employ a combination of gene knockout models and *in vitro* cellular systems, applying diverse modeling strategies to elucidate the specific therapeutic mechanisms of TCM across multiple biological levels. (3) In terms of stimulus selection, it would be more clinically relevant to use stimulants associated with UC pathology, such as patient-derived serum or cytokine mixtures representative of UC, with PMA serving as a positive control (Boeltz et al., 2019). (4) Causal relationships between microbiota and NETs remain unclear in many studies, with some only demonstrating correlations. Antibiotic-induced microbiota depletion or FMT is strongly recommended in these studies. The precise mechanisms underlying the interaction between NETs and microbiota have yet to be fully elucidated. Future research should aim to conduct more in-depth and clear experiments in this area. (6) The bioavailability of phytochemicals derived from TCM is often constrained by their limited chemical stability and aqueous solubility. This challenge can be addressed through the design of novel delivery platforms (nanocarriers, lipid-based vesicles, polymeric gels, etc.), which significantly improve their absorption and bioavailability.

## 6 Discussion

UC prominently features neutrophil-driven inflammation. While initial therapeutic advances focus on restraining this immune cell activity, indiscriminate neutrophil depletion is counterproductive. These cells serve as vital defenders, safeguarding intestinal balance, combating pathogens, and facilitating wound repair and homeostatic maintenance. Studies on NETs offer a more targeted strategy. Removing NETs disrupts pathological processes without terminating core neutrophil functions. Crucially, NET formation inhibition breaks the self-perpetuating inflammatory cycle while preserving essential antimicrobial defenses. Moreover, microbiota is considered both as a causal factor and a consequence of the disease process, thereby forming a central link within the pathogenic cycle. Critically, the dynamic and reciprocal interactions between the GM and the host immune defense are now understood to be key interconnected drivers that perpetuate intestinal inflammation and contribute significantly to tissue damage in UC.

Our review highlights the efficacy of TCM in suppressing NET formation and modulating GM during UC. Several herbs and formulas demonstrably lower levels of key NET constituents, including NE, MPO, and citH3. These interventions concurrently reduce ROS generation and inhibit PAD4 activity, effectively antagonizing NETosis. Further research demonstrates that TCM therapies modulate critical inflammatory signaling cascades (e.g., MAPK, NF-κB) and oxidative stress pathways (e.g., Nrf2, HIF-1α). Additionally, TCM agents disrupt exosome-facilitated communication between neutrophils and epithelial cells, collectively resulting in diminished neutrophil activation. Moreover, a significant body of research has linked NET modulation with the microbiota. The anti-inflammatory effects of certain herbs and formulas are microbiota-dependent. TCM derivatives regulate not only the diversity and composition but also the function of gut flora, typically exerting a bidirectional effect that restores beneficial probiotics while reducing pathogenic microbes disrupted in UC. These studies underscore how TCM’s holistic approach achieves comprehensive efficacy by targeting the complex UC network, rather than focusing on isolated targets, offering a unique advantage of TCM in addressing the multifaceted nature of UC.
